# Population pharmacokinetics modelling to predict DDI from zopiclone on clozapine in schizophrenia patients

**DOI:** 10.3389/fpsyt.2025.1664678

**Published:** 2025-09-22

**Authors:** Huan-Huan Han, Yue Zhang, Jie Wang, Xue Tian, Ye Li, Su-Mei He, Cun Zhang, Xiao Chen, Dong-Dong Wang

**Affiliations:** ^1^ Department of Pharmacy, The Affiliated Lianyungang Hospital of Xuzhou Medical University, Lianyungang, Jiangsu, China; ^2^ Jiangsu Key Laboratory of New Drug Research and Clinical Pharmacy & School of Pharmacy, Xuzhou Medical University, Xuzhou, Jiangsu, China; ^3^ Department of Pharmacy, Suzhou Research Center of Medical School, Suzhou Hospital, Affiliated Hospital of Medical School, Nanjing University, Suzhou, Jiangsu, China; ^4^ Department of Pharmacy, Xuzhou Oriental Hospital Affiliated to Xuzhou Medical University, Xuzhou, Jiangsu, China; ^5^ School of Nursing, Xuzhou Medical University, Xuzhou, Jiangsu, China

**Keywords:** population pharmacokinetics modelling, drug-drug interactions, individualized therapy, clozapine, schizophrenia

## Abstract

**Introduction:**

Clozapine, as a core drug for the treatment of schizophrenia, is widely used in the drug treatment of schizophrenia patients. However, when multiple drugs are used in combination, it is not clear whether there are drug-drug interactions (DDI) of clozapine in patients with schizophrenia. This study aims to use population pharmacokinetics (PPK) modelling to predict DDI and individualized therapy of clozapine in schizophrenia patients.

**Methods:**

We collected 81 patients with schizophrenia and included their physiological data, biochemical data, treatment plans and information on combined medication during the clinical treatment process. Next, PPK modelling was used to analyze drugs with potential DDI when clozapine was used in schizophrenia patients, and dosage adjustments were recommended.

**Results:**

Final analysis revealed that weight and coadministration of zopiclone affected clozapine clearance, and there was DDI with clozapine when zopiclone was used concurrently in schizophrenia patients. Further, for schizophrenia patients without zopiclone, 10 mg/kg/day, 9 mg/kg/day, 8 mg/kg/day and 7 mg/kg/day clozapine were recommended for 40–50 kg, 50–67 kg, 67–88 kg, and 88–120 kg patients, respectively. For schizophrenia patients with zopiclone, 6 mg/kg/day and 5 mg/kg/day clozapine were recommended for 40–70 kg and 70–120 kg patients, respectively. This study was the first to systematically analyze DDI when clozapine was used in schizophrenia patients and found DDI when zopiclone and clozapine were taken concurrently.

**Conclusion:**

When zopiclone was taken concurrently, clozapine dosage need to be reduced. Based on this, schizophrenia patients individualized dosage adjustment was recommended.

## Introduction

Schizophrenia is a psychotic disorder characterized by a combination of positive and negative symptoms, along with cognitive impairment, affective symptoms, and behavioral disturbances (1). In terms of epidemiology, schizophrenia has a lifetime prevalence of 1% (2, 3), and the incidence is high among young and middle-aged people, the sex ratio is close, the genetic factors are significant, and the risk of developing schizophrenia is significantly increased when the immediate family member is schizophrenia.

The mainstay of schizophrenia treatment is the use of atypical antipsychotic medications, and non-pharmacological treatment serves as a complementary therapy, where clozapine, as the core drug for treating schizophrenia, occupies an important position in the drug treatment of schizophrenia patients, especially suitable for refractory cases (4, 5). Clozapine is not only particularly useful in treatment-resistant schizophrenia as monotherapy, but it is also used in combination therapy, such as combination with aripiprazole, lurasidone, or cariprazine (6–13).

It is important that latest research indicating that the use of clozapine at recommended doses does not guarantee achieving therapeutic concentrations of clozapine and women and nonsmokers were at the highest risk of having toxic levels of clozapine (14). Drug-drug interactions (DDI) can significantly affect drug concentrations, and the occurrence of DDI is often accompanied during the treatment of mental disorders (15, 16). For example, zopiclone is metabolized by the CYP3A4 enzyme (17, 18), which may compete CYP3A4 metabolic enzymes with clozapine, influence clozapine clearance in schizophrenia patients. In the routine clinical practice, the dosage information for both clozapine and zopiclone are mainly based on the instructions.

Population pharmacokinetics (PPK), by integrating sparse clinical data with covariate modelling, addresses the limitations of traditional pharmacokinetics in real-world complex populations. Its core value lies in quantifying sources of variation (such as differences in liver and kidney functions), providing feasible pharmacokinetic research methods for special populations (children, the elderly), and identifying key influencing factors (especially DDI), supporting precision medical decision-making (19). At present, PPK has been widely used in the analysis of potential DDI in clinical practice. For example, Fujita et al. reported PPK Analysis of DDI between perampanel and carbamazepine using enzyme induction model in epileptic patients (20). Cleary et al. reported estimation of FMO3 ontogeny by mechanistic PPK modelling of risdiplam and its impact on DDI in children (21). Li et al. reported PPK of ruxolitinib in children with hemophagocytic lymphohistiocytosis: focus on the DDI (22). Courlet et al. reported PPK modelling to quantify the magnitude of DDI between amlodipine and antiretroviral drugs (23). Barcelo et al. reported PPK of dolutegravir: influence of DDI in a real-life setting (24).

Therefore, this study aims to using PPK modelling to predict DDI of clozapine in schizophrenia patients, and to recommend individualized dosage adjustments for these patients.

## Methods

### Data collection

This study was approved by the Research Ethics Committee of Xuzhou Oriental Hospital Affiliated to Xuzhou Medical University, which collected schizophrenia patients at Xuzhou Oriental Hospital Affiliated to Xuzhou Medical University from December 2023 to November 2024, including their physiological data, biochemical data, treatment plans and information on combined medication during the clinical treatment process, where the requirement for written informed consent could be waived since the data were collected retrospectively without patient identifiers. The dosage information for clozapine was mainly based on the instruction. The analytical technique used for the determination of clozapine was homogeneous enzyme immunoassay. The sample extraction times for plasma concentrations were before the next administration, which was the value of the trough concentration.

### Model building

PPK model of clozapine in schizophrenia patients was set up, where CL/F, V/F, and Ka [fixed at 1.3/h (25, 26)] were the main pharmacokinetic parameters. In terms of individual variation, we chose to express it using Equation 1:


(1) $${\text{Z}}_{\text{i}}=\text{TV}\left(\text{Z}\right)\times \text{exp }\left(\eta_{i}\right)$$

The abbreviation Z_i_ denoted individual parameter, TV(Z) denoted typical individual parameter and η_i_ denoted symmetrical distribution.

In terms of random residual variation, we chose to express it using Equation 2:


(2) $${\text{Y}}_{\text{i}}=\;{\text{X}}_{\text{i}}+\;{\text{X}}_{\text{i}*}{\text{ϵ}}_{1}$$

The abbreviation Y_i_ denoted observed concentration, X_i_ denoted individual predicted concentration, and ϵ_1_ denoted symmetrical distribution.

In terms of relationship between parameter and weight, we chose to express it using Equation 3:


(3) $${\text{U}}_{\text{i}}={\text{U}}_{\text{std}}\times {\left({\text{V}}_{\text{i}}/{\text{V}}_{\text{std}}\right)}^{\text{W}}$$

The abbreviation U_i_ denoted individual parameter, V_i_ denoted individual weight, V_std_ denoted standard weight of 70 kg, and U_std_ denoted typical individual parameter. W denoted allometric coefficients: 0.75 and 1 for CL/F and V/F, respectively (27).

In terms of continuous or categorical covariate parameter, we chose to express it using Equation 4 or 5, respectively:


(4) $${\text{R}}_{\text{i}}=\text{TV}\left(\text{R}\right)\times {\left({\text{S}}_{\text{i}}/{\text{S}}_{\text{m}}\right)}^{\text{Q}}$$

(5) $${\text{R}}_{\text{i}}=\text{TV}\left(\text{R}\right)\times (1+\text{Q}\times {\text{S}}_{\text{i}})$$

The abbreviation R_i_ denoted individual parameter, TV(R) denoted typical individual parameter, Q denoted the parameter needed to be fitted, S_i_ denoted covariate of the i-th individual, and S_m_ denoted population median for the covariate. To construct covariate model, we used two-step method.

### Model evaluation

We used visual diagram and bootstrap methods to evaluate the final clozapine PPK model of schizophrenia patients.

### Dosage simulation

We used Monte Carlo simulation to simulate the clozapine concentrations of schizophrenia patients under different simulated clozapine dosages, including 1 mg/kg/day, 2 mg/kg/day, 3 mg/kg/day, 4 mg/kg/day, 5 mg/kg/day, 6 mg/kg/day, 7 mg/kg/day, 8 mg/kg/day, 9 mg/kg/day, 10 mg/kg/day. Additionally, the simulated patients were divided into two parts: (a) schizophrenia patients not taking zopiclone, and (b) schizophrenia patients taking zopiclone, where simulated weight groups contained 40 kg, 60 kg, 80 kg, 100 kg, 120 kg. We simulated each scenario 1000 times, and the therapeutic range was 350–800 ng/ml along with 1000 ng/ml toxicity threshold (28–31).

## Results

### Patient’s data

We collected 81 patients with schizophrenia. 37 schizophrenia patients were men and 44 schizophrenia patients were women. The age ranges were from 20.67 to 73.11 years old, and weight ranges were from 38.00 to 120.00 kg. **Tables 1** and **2** denoted demographic data of schizophrenia patients and drug combination, respectively.

**Table 1:** Schizophrenia patients (n = 81).

Characteristic	Mean ± SD	Median (range)
Gender (men/women)	37/44	/
Age (years)	49.46 ± 11.15	50.67 (20.67-73.11)
Weight (kg)	70.49 ± 13.53	71.00 (38.00-120.00)
Albumin (g/L)	39.46 ± 3.21	39.40 (27.90-47.90)
Globulin (g/L)	26.28 ± 3.23	26.30 (19.60-35.10)
Alanine transaminase (IU/L)	25.47 ± 21.61	20.00 (4.00-162.00)
Aspartate transaminase (IU/L)	21.46 ± 12.58	19.00 (9.00-119.00)
Creatinine (μmol/L)	61.14 ± 11.37	60.00 (32.00-96.00)
Urea (mmol/L)	4.54 ± 1.39	4.33 (1.82-11.71)
Total protein (g/L)	65.74 ± 4.71	66.30 (51.90-76.60)
Total cholesterol (mmol/L)	4.08 ± 0.85	4.04 (2.27-6.51)
Triglyceride (mmol/L)	1.58 ± 0.81	1.45 (0.44-5.11)
Direct bilirubin (μmol/L)	2.33 ± 1.18	2.00 (0.50-8.30)
Total bilibrubin (μmol/L)	7.35 ± 3.27	6.60 (2.70-21.30)
Hematocrit (%)	37.98 ± 3.52	37.40 (31.40-49.20)
Hemoglobin (g/L)	124.93 ± 14.53	124.00 (21.00-166.00)
Mean corpuscular hemoglobin (pg)	30.17 ± 1.45	30.40 (24.50-34.00)
Mean corpuscular hemoglobin concentration (g/L)	329.93 ± 7.89	330.00 (312.00-351.00)

**Table 2:** Drug combination (n = 81).

Drug	Category	N	Drug	Category	N
Acarbose capsules	0	75	Metoprolol succinate sustained-release tablets	0	78
	1	6		1	3
Alprazolam tablets	0	76	Nifedipine sustained-release tablets	0	78
	1	5		1	3
Amisulpride tablets	0	71	Paliperidone sustained-release tablets	0	77
	1	10		1	4
Amlodipine besylate tablets	0	78	Perphenazine tablets	0	79
	1	3		1	2
Aripiprazole tablets	0	60	Phenhyxol hydrochloride tablets	0	62
	1	21		1	19
Atorvastatin calcium tablets	0	75	Propranolol hydrochloride tablets	0	63
	1	6		1	18
Bezafibrate	0	79	Risperidone oral liquid	0	79
	1	2		1	2
Clonazepam tablets	0	72	Risperidone tablets	0	53
	1	9		1	28
Enteric-coated aspirin tablets	0	77	Sertraline hydrochloride tablets	0	77
	1	4		1	4
Glimepiride	0	79	Sodium valproate sustained-release tablets	0	59
	1	2		1	22
Lamotrigine tablets	0	79	Sulpiride tablets	0	80
	1	2		1	1
Lithium carbonate extended-release tablets	0	72	Valsartan capsules	0	76
	1	9		1	5
Lorazepam tablets	0	70	Ziprasidone hydrochloride capsules	0	75
	1	11		1	6
Metformin hydrochloride tablets	0	68	Zopiclone tablets	0	73
	1	13		1	8

Category, 0: without drug, 1: with drug; N: number of patients.

### PPK modelling

Equations 6 and 7 were the final clozapine PPK model of schizophrenia patients:


(6) $$\text{CL}/\text{F}=29.6\times {\left(\text{weight}/70\right)}^{0.75}\times \left(1-0.254\times \text{ZOP}\right)$$

(7) V/F=308×(weight/70)

ZOP denoted zopiclone and when schizophrenia patients took ZOP, ZOP denoted 1, otherwise ZOP denoted 0.

### Model evaluation

**Figure 1:**
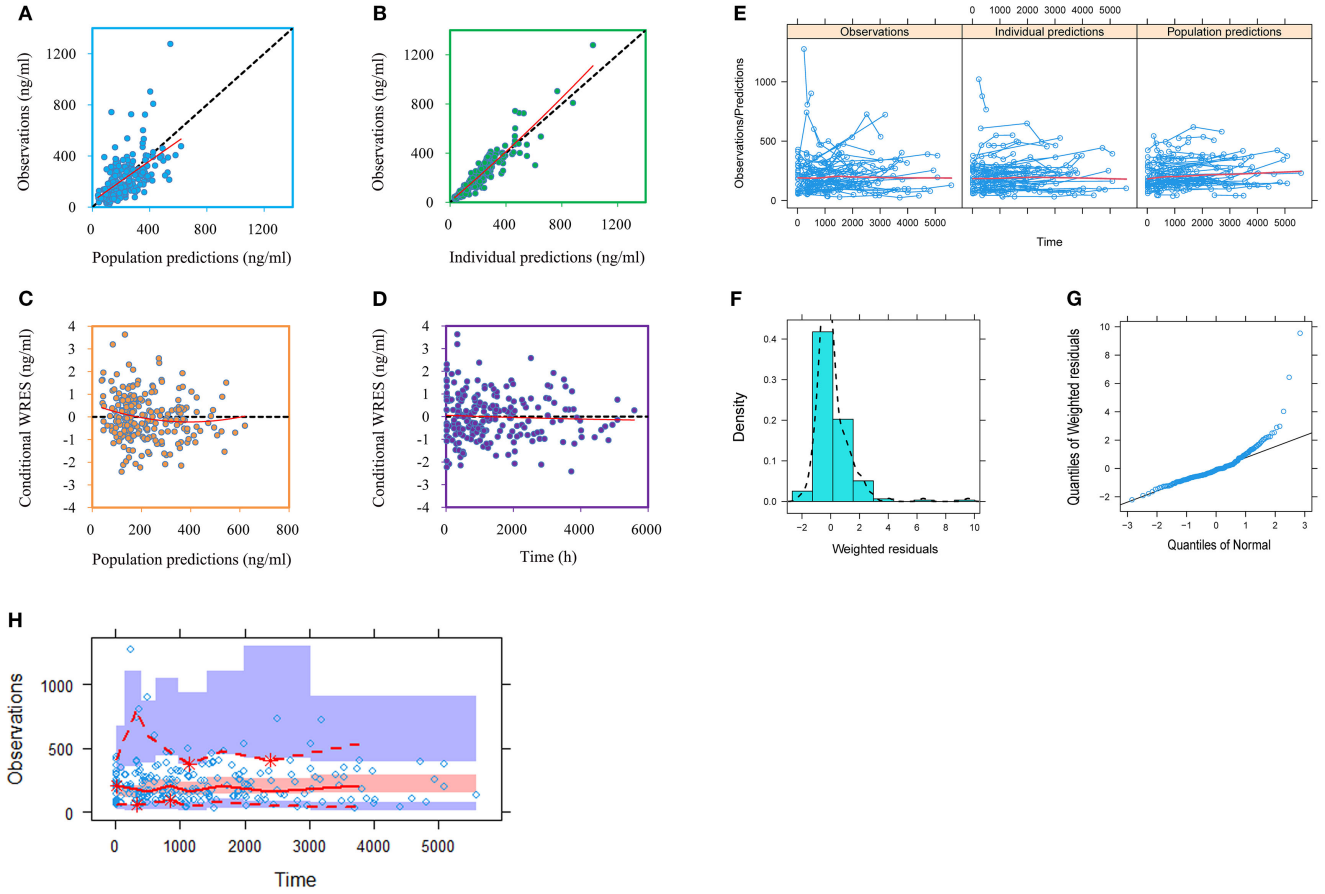
Model evaluation. **(A)** Observations *vs*. population predictions. **(B)** Observations *vs*. individual predictions. **(C)** Conditional weighted residuals (WRES) *vs*. population predictions. **(D)** Conditional WRES *vs.* time. **(E)** Observations/Predictions *vs.* time. **(F)** Density *vs*. weighted residuals. **(G)** Quantilies of weighted residuals *vs*. quantilies of normal. **(H)** Visual predictive check (VPC) of model.

**Figure 2:**
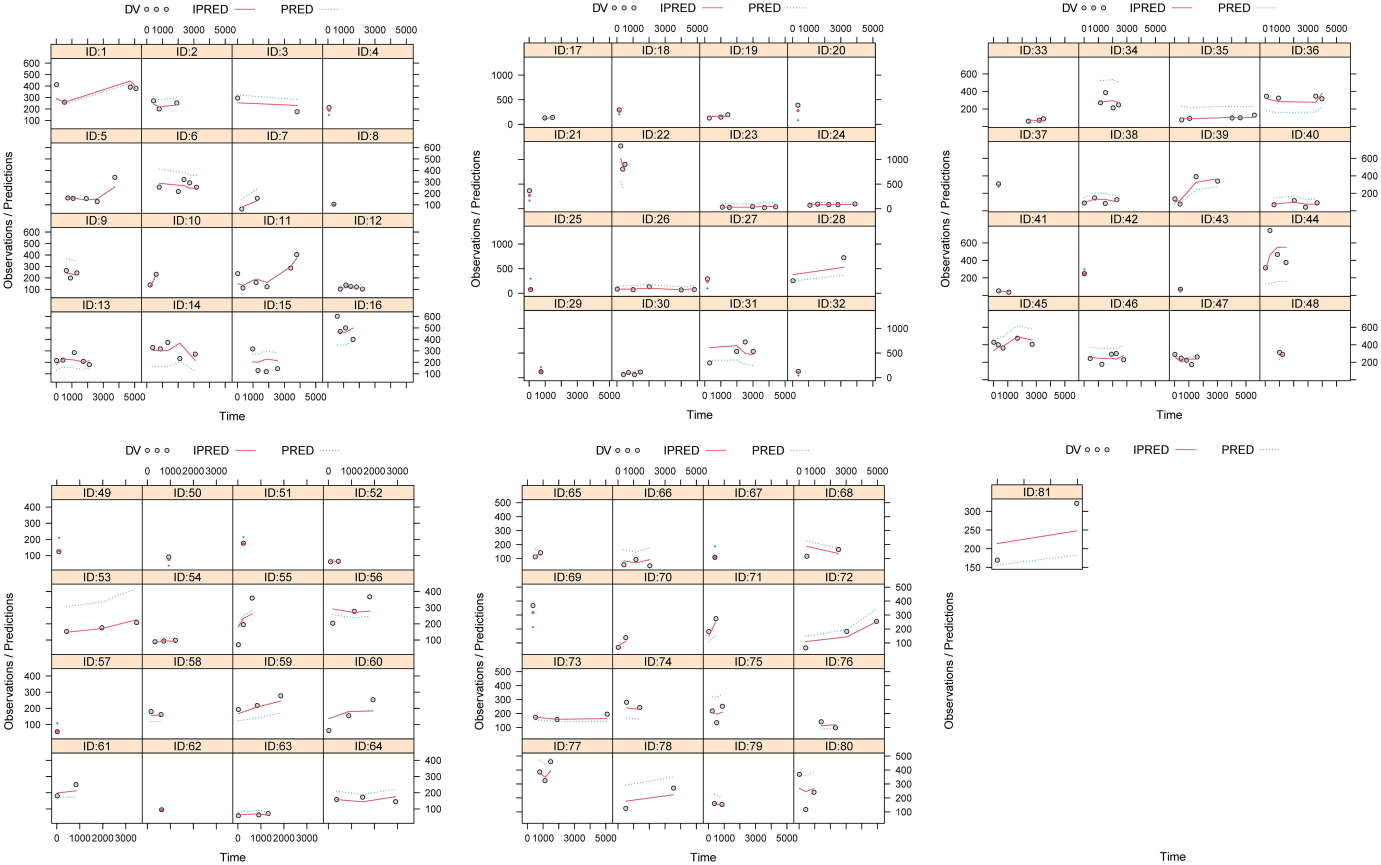
Individual plots.

**Figure 3:**
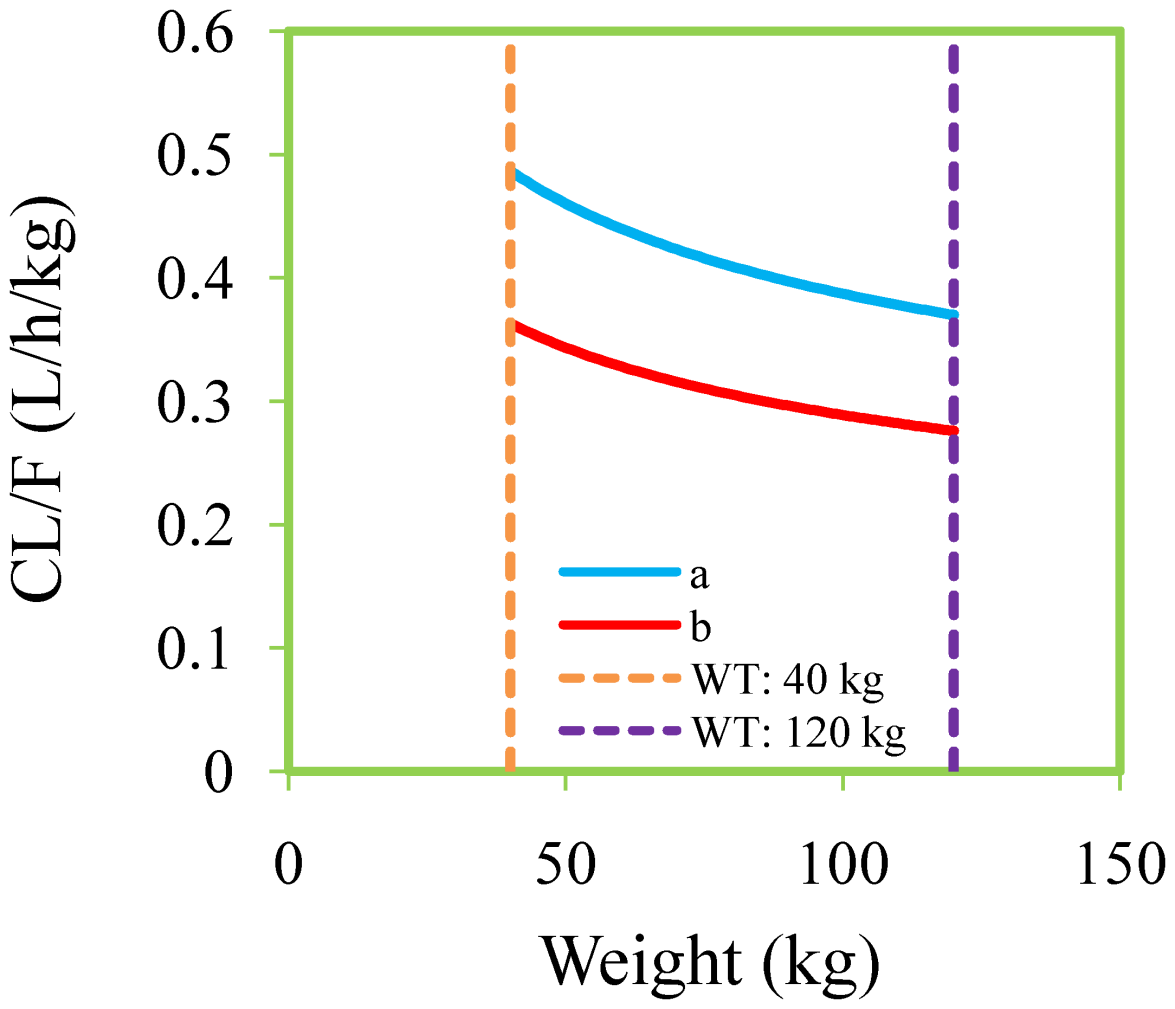
Clozapine clearance rates of schizophrenia patients. **(A)** Schizophrenia patients not taking zopiclone. **(B)** Schizophrenia patients taking zopiclone.

**Figures 1**, **2** and **Table 3** denoted visual diagram, individual plots and bootstrap, showing clozapine PPK model of schizophrenia patients was credible. When schizophrenia patients taking zopiclone, the clozapine clearance of schizophrenia patients was reduced by 25.4%, which was shown in **Figure 3**.

**Table 3:** Parameter estimates of clozapine final model and bootstrap validation in schizophrenia patients.

Parameter	Estimate	SE (%)	Bootstrap			Bias (%)
			Median	95% Confidence interval	SE(%)	
CL/F (L/h)	29.6	6.5	29.4	[26.1, 33.9]	6.5	-0.68
V/F (L)	308	14.4	309	[230, 421]	14.4	0.32
Ka (h^-1^)	1.3 (fixed)	–	–	–	–	–
θ_ZOP_	-0.254	30.8	-0.241	[-0.408, -0.009]	30.4	-5.12
ω_CL/F_	0.348	11.3	0.342	[0.264, 0.422]	11.0	-1.72
σ_1_	0.257	6.6	0.254	[0.220, 0.289]	6.6	-1.17

95% confidential interval was displayed as the 2.5th, 97.5th percentile of bootstrap estimates. CL/F, apparent oral clearance (L/h); V/F, apparent volume of distribution (L); Ka, absorption rate constant (h^-1^); θ_ZOP_ was the coefficient of zopiclone; ω_CL/F_, inter-individual variability of CL/F; σ_1_, residual variability, proportional error; Bias, prediction error, Bias = (Median-Estimate)/Estimate×100%.

### Dosage recommendation and safety evaluation

**Figure 4:**
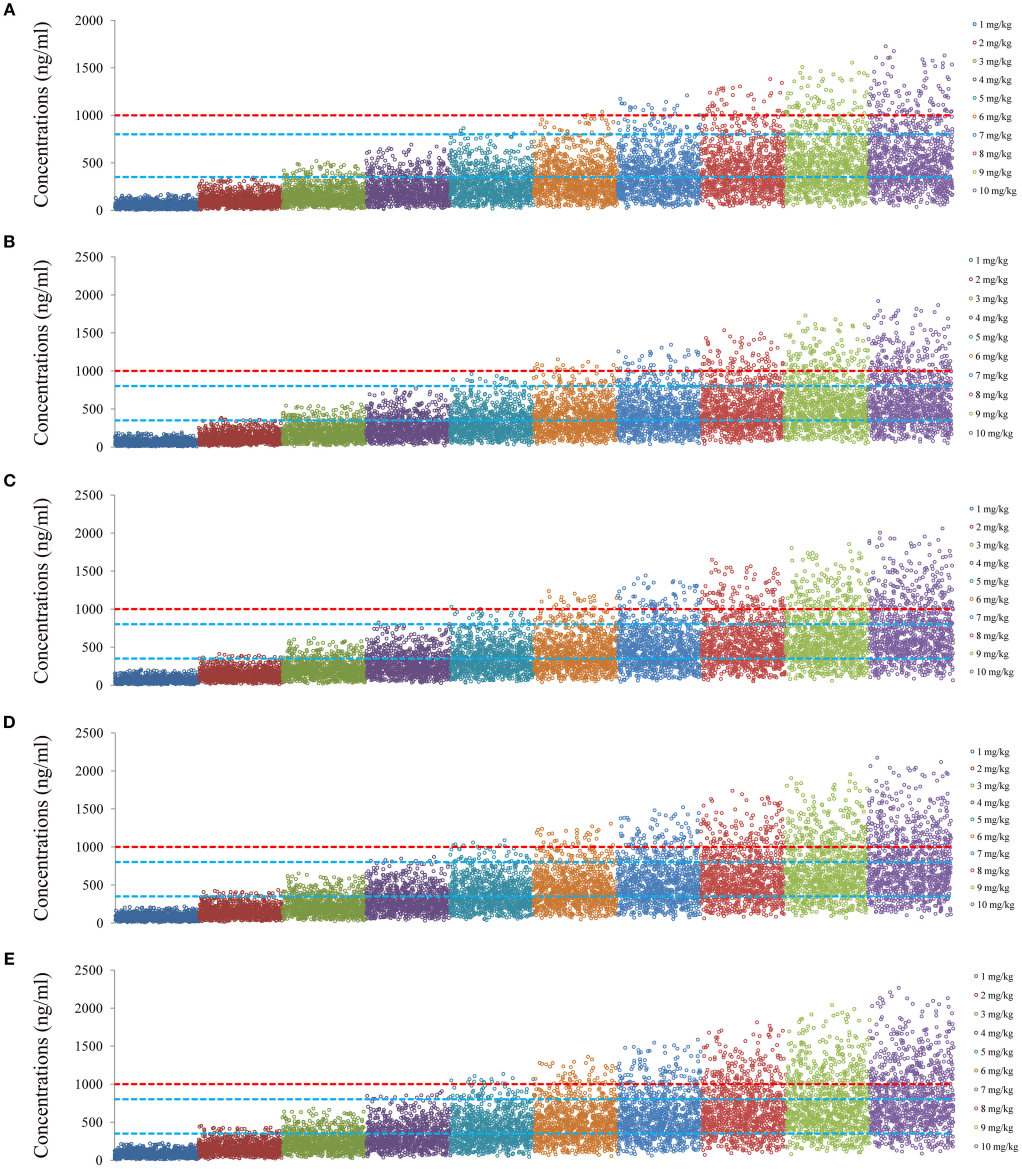
Simulated clozapine concentrations of schizophrenia patients not taking zopiclone. **(A)** 40 kg schizophrenia patients. **(B)** 60 kg schizophrenia patients. **(C)** 80 kg schizophrenia patients. **(D)** 100 kg schizophrenia patients. **(E)** 120 kg schizophrenia patients.

**Figure 5:**
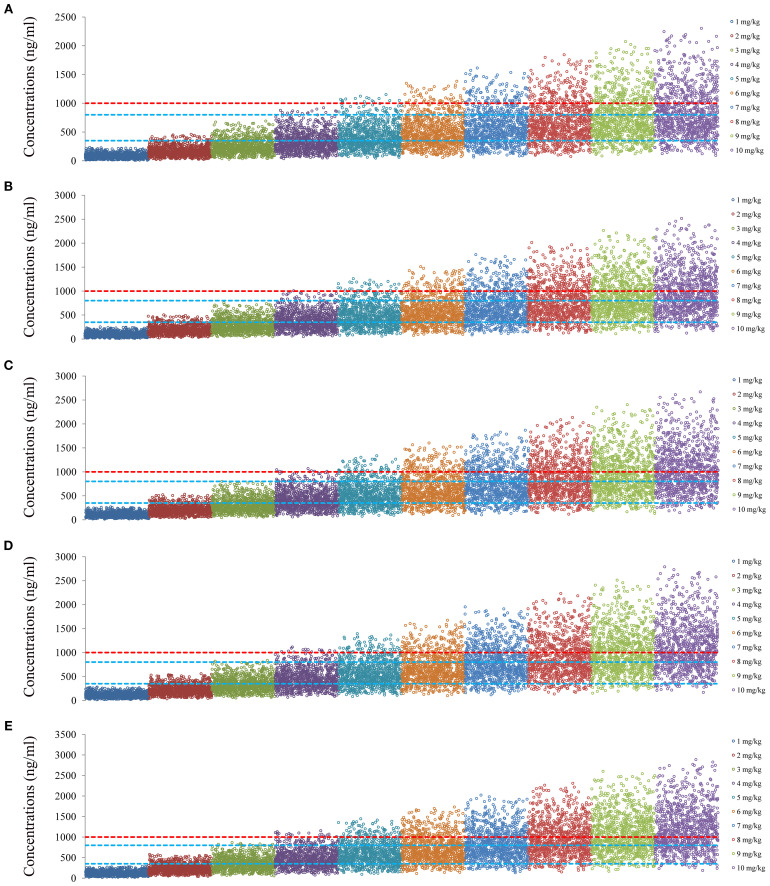
Simulated clozapine concentrations of schizophrenia patients taking zopiclone. **(A)** 40 kg schizophrenia patients. **(B)** 60 kg schizophrenia patients. **(C)** 80 kg schizophrenia patients. **(D)** 100 kg schizophrenia patients. **(E)** 120 kg schizophrenia patients.

**Figure 6:**
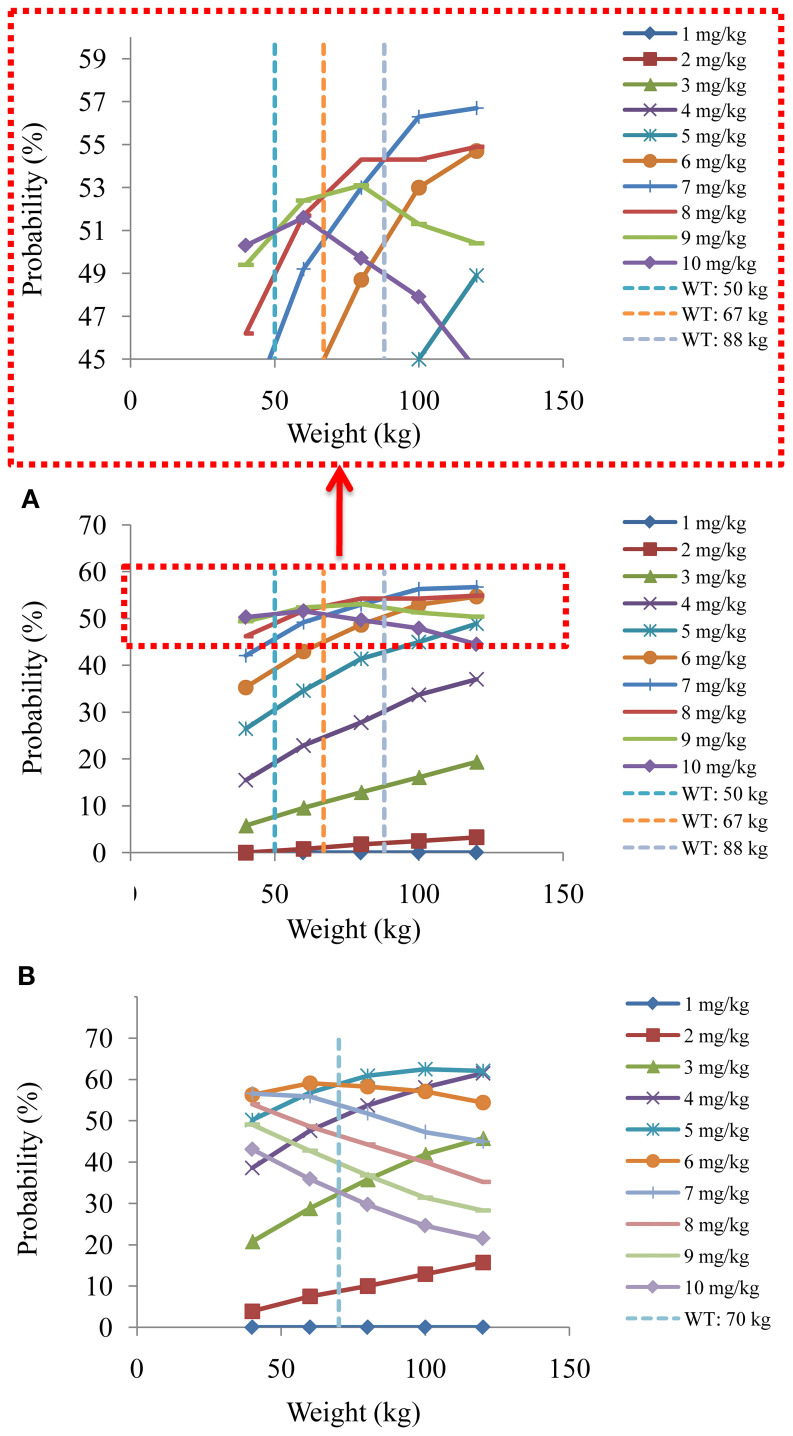
The probabilities to attain the target clozapine concentrations of schizophrenia patients. **(A)** Schizophrenia patients not taking zopiclone. **(B)** Schizophrenia patients taking zopiclone.

**Figure 7:**
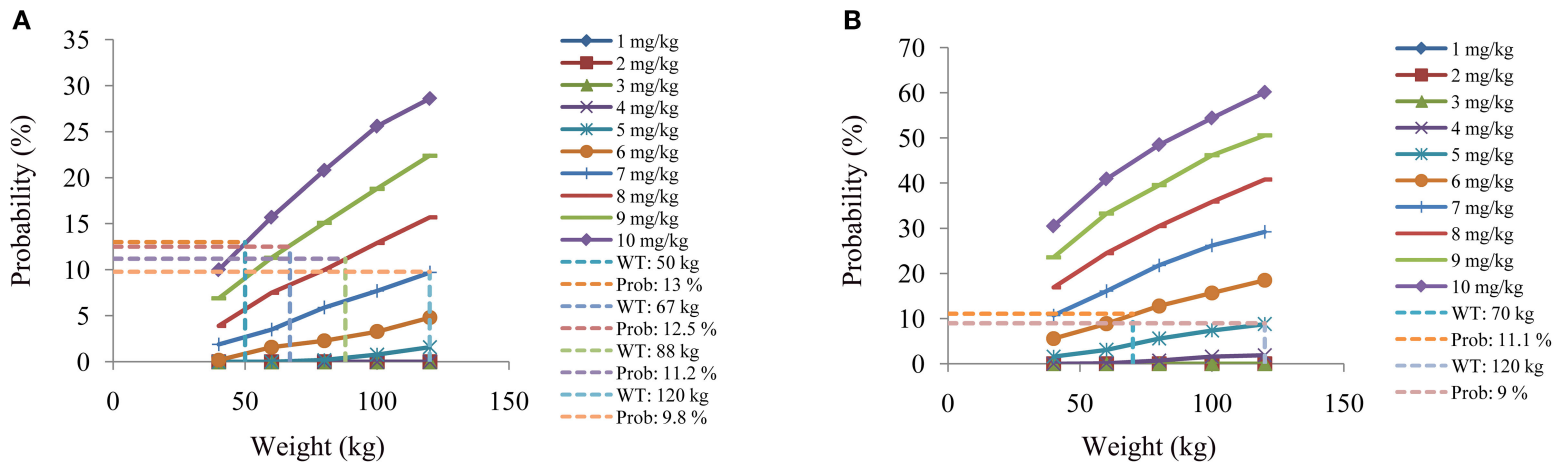
The probabilities to exceed the upper limit of safe concentrations of schizophrenia patients. **(A)** Schizophrenia patients not taking zopiclone. **(B)** Schizophrenia patients taking zopiclone.

**Figures 4** and **5** denoted simulated clozapine concentrations of schizophrenia patients not taking zopiclone and simulated clozapine concentrations of schizophrenia patients taking zopiclone, respectively. **Figure 6** denoted the probabilities to attain the target clozapine concentrations of schizophrenia patients. For schizophrenia patients without zopiclone, 10 mg/kg/day, 9 mg/kg/day, 8 mg/kg/day and 7 mg/kg/day clozapine were recommended for 40–50 kg, 50–67 kg, 67–88 kg, and 88–120 kg patients, respectively. For schizophrenia patients with zopiclone, 6 mg/kg/day and 5 mg/kg/day clozapine were recommended for 40–70 kg and 70–120 kg patients, respectively. **Figure 7** denoted the probabilities to exceed the upper limit of safe concentrations of schizophrenia patients. The detailed dosage recommendation and safety evaluation were shown in **Table 4**.

**Table 4:** Initial dosage recommendation of clozapine in schizophrenia patients.

Without zopiclone			With zopiclone		
Body weight (kg)	Dosage (mg/kg/day)	Probability to exceed the upper limit of the safe concentration (%)	Body weight (kg)	Dosage (mg/kg/day)	Probability to exceed the upper limit of the safe concentration (%)
[40-50)	10	< 13	[40-70)	6	< 11.1
[50-67)	9	< 12.5	[70-120]	5	< 9
[67-88)	8	< 11.2			
[88-120]	7	< 9.8			

## Discussion

Clozapine is a classic old drug that was synthesized over 65 years ago, which is the only psychotherapeutic drug been used to treat treatment-resistant schizophrenia. up to now, the actual mechanism why clozapine has a remarkable therapeutic effect in the treatment of treatment-resistant schizophrenia (32). Regarding when to initiate clozapine therapy for patients with schizophrenia remains contentious. According to current guidelines, clozapine is recommended for initiation when patients show no response after two adequate courses of treatment with standard antipsychotic drugs (32). However, recent research indicates that in patients with first-episode schizophrenia experiencing their first psychotic relapse, neither continuing the same non-clozapine oral antipsychotic nor switching to another non-clozapine oral antipsychotic demonstrated beneficial effects for relapse prevention (33). These findings, combined with existing knowledge regarding clozapine’s association with reduced mortality, challenge current treatment guidelines recommending clozapine as a third-line therapy (33). That is to say, the earlier clozapine is used among patients with schizophrenia, the more benefits they may gain.

However, patients with schizophrenia often have more concomitant medications. In the clinical application process, combined medication often needs to be viewed dialectically. It is a double-edged sword. Reasonable application can significantly enhance the therapeutic effect, but unreasonable use may cause serious risks. Especially when combined medication causes bad DDI, it may pose a serious threat to the efficacy of the drug and even the health of the patient. When the harmful drug significantly increases or decreases the clearance rate of the victimized drug, and thereby significantly reduces or increases the blood concentration of the target drug, it is necessary to focus on pharmaceutical care, and even a new administration plan for the target drug needs to be reformulated.

These DDI clinical cases include the use of immunosuppressants after organ transplantation (34–38), clinical use of hypoglycemic drugs (39–41), individualized administration of anti-hepatitis virus drugs (42, 43), clinical use of anticoagulant drugs (44–46), individualized drug administration for patients with dementia (47), clinical medication for patients with asthma (48), individualized drug administration for patients with liver and kidney damage (49, 50), rational drug use for patients with heart failure (51), individualized drug administration for patients with atrial fibrillation (52), clinical medication for patients with HIV (53–56), precise medication for special pediatric populations (57, 58), individualized drug administration for the special population of pregnant women (59, 60), use of antibiotics for critically infected patients in the ICU (61), clinical medication for COVID-19 patients (62), clinical use of anti-tumor drugs in cancer patients (63–71), clinical use of pain treatment drugs for cancer patients (72), which have been widely carried out.

Based on this, in the current research, we used PPK modelling to predict DDI of clozapine in schizophrenia patients. This study included the drugs used in combination in the schizophrenia population, including acarbose capsules, alprazolam tablets, amisulpride tablets, amlodipine besylate tablets, aripiprazole tablets, atorvastatin calcium tablets, bezafibrate, clonazepam tablets, enteric-coated aspirin tablets, glimepiride, lamotrigine tablets, lithium carbonate extended-release tablets, lorazepam tablets, metformin hydrochloride tablets, metoprolol succinate sustained-release tablets, nifedipine sustained-release tablets, paliperidone sustained-release tablets, perphenazine tablets, phenhyxol hydrochloride tablets, propranolol hydrochloride tablets, risperidone oral liquid, risperidone tablets, sertraline hydrochloride tablets, sodium valproate sustained-release tablets, sulpiride tablets, valsartan capsules, ziprasidone hydrochloride capsules, zopiclone tablets.

Finally, the PPK model found that weight and coadministration of zopiclone affected the clearance rate of clozapine, and there was DDI with clozapine when zopiclone was used concurrently in schizophrenia patients. When schizophrenia patients took zopiclone simultaneously, the clozapine clearance rate of the patients decreased by 25.4%. This is mainly because clozapine is mainly metabolized in the liver through CYP3A4 and CYP1A2 (4, 73, 74), and zopiclone is also metabolized by the CYP3A4 enzyme (17, 18), which may compete CYP3A4 metabolic enzymes with clozapine, influence clozapine clearance in schizophrenia patients. Furthermore, we optimized the optimal dosage adjustment of clozapine in schizophrenia patients with or without zopiclone through Monte Carlo simulation. For schizophrenia patients without zopiclone, 10 mg/kg/day, 9 mg/kg/day, 8 mg/kg/day and 7 mg/kg/day clozapine were recommended for 40–50 kg, 50–67 kg, 67–88 kg, and 88–120 kg patients, respectively. For schizophrenia patients with zopiclone, 6 mg/kg/day and 5 mg/kg/day clozapine were recommended for 40–70 kg and 70–120 kg patients, respectively.

## Conclusion

This study was the first to systematically analyze DDI when clozapine was used in schizophrenia patients and found DDI when zopiclone and clozapine were taken concurrently. Furthermore, when zopiclone was taken concurrently, the requirement of clozapine dosage needed to reduce. Based on this, schizophrenia patients individualized dosage adjustment was recommended.

## Data Availability

The original contributions presented in the study are included in the article/supplementary material. Further inquiries can be directed to the corresponding author/s.
